# The effects of handling on mouse behavior: cupped hands versus familiar or novel huts or tunnels

**DOI:** 10.1371/journal.pone.0323785

**Published:** 2025-05-19

**Authors:** Maya J. Bodnar, I. Joanna Makowska, Catherine A. Schuppli, Daniel M. Weary

**Affiliations:** Animal Welfare Program, Faculty of Land and Food Systems, University of British Columbia, Vancouver, Canada; Tokai University School of Medicine, JAPAN

## Abstract

Mice are commonly tail-handled, despite evidence that this is aversive. Alternatives include cupping and tunnel handling; both methods are associated with improved welfare outcomes, including reduced anxiety and improved ease of handling, but tail handling may be perceived as more practical for handlers. Practicality may be improved by using handling objects already present in facilities, such as upturned mouse huts. Our first aim was to compare hut handling with the established refined alternatives of cupping and tunnel handling. As both tunnels and huts may be used as part of mouse caging, a second aim was to assess the effects of handling object familiarity (from the home cage vs. a novel object). Outcomes assessed were voluntary interaction with the handler and time spent in the open arms of an elevated plus maze (EPM). Mice (n = 51) were randomly assigned a handling method: cupping, tunnel, or hut. Cages (n = 14) were randomly assigned to contain either a tunnel or hut. Mice underwent 9 days of handling and voluntary interaction tests were conducted on days 1, 5, and 9. On day 10, mice were tested in the EPM. We found that interaction varied with handling object: hut-handled mice spent the most time interacting, followed by tunnel-handled and cupped mice (41.7 ± 1.5 s, 36.1 ± 1.4 s, and 33.0 ± 1.5 s, respectively). Familiar objects improved interaction at the outset, but this difference was no longer evident by day 5. We found no effect of handling object or object familiarity on time spent in the open arms of the EPM. These results suggest that hut handling is a refined handling method; this method may be a practical alternative in facilities that already use huts.

## Introduction

Mice used in laboratories are often subject to handling during routine husbandry events and experimental procedures. Mice are commonly handled by their tail (i.e., picked up by grasping the base of the tail between the thumb and forefinger), despite evidence that tail handling is aversive [1–5]. In addition to the negative effects on mouse welfare, the stress associated with tail handling can introduce variability to data, reducing the reliability of findings from these studies [6,7].

Refined handling methods include cupping (i.e., scooping mice into open cupped hands) and tunnel handling (i.e., gently guiding mice into a handling tunnel). Compared to tail handling, these refinements are associated with behavioral and physiological changes consistent with improved mouse welfare, including more sucrose consumption in the sucrose consumption test [4,8], improved breeding success [9], and reduced blood corticosterone [3]. Use of refined handling methods is also associated with reduced pain grimace scores [10] and continued positive interactions with experimenters following aversive procedures [1,5,11,12], resulting in mice who are easier to handle [5]. There is also some evidence that familiarization with the handling object (e.g., tunnel) can improve responses to handling in some mice [2].

Despite the benefits of refined handling, many laboratories continue to use tail handling, perhaps because of various negative perceptions of refined methods, including the cost of purchasing tunnels, that it will take more time to handle animals, and that common procedures will be more difficult to perform [13–15]. Cupping presents additional challenges, such as variability in its effects on mouse behavior [16], longer acclimatization period [1,11], the perceived risk of bites from mice, and biosecurity hazards [15]. These barriers to adopting cupping and tunnel handling suggest the need to explore other refined handling methods.

Objects already available within mouse cages are promising candidates for handling because they are readily available and familiar with the animals. For example, plastic cage ladders have been used for handling mice, and this method resulted in less anxiety compared to tail handling [17]. Other materials commonly available in mouse cages include shelters or huts; some researchers have reported using upturned huts to handle mice (e.g., [18,19]), and the results of one student thesis suggest that, compared to tail-handled mice, hut-handled mice display less anxiety and more voluntary interaction with the handler [20]. To date, no work has compared hut handling with the gold standard of tunnel handling [16] and cupping.

Our study aimed to assess the effects of handling method (cupping, tunnel handling, or hut handling) on voluntary interaction with the handler and/or handling object (hereafter referred to as ‘handler’) and anxiety, as assessed by the elevated plus maze. As both tunnels and huts may be used as part of mouse caging, a second aim was to assess the effects of handling object familiarity (i.e., always present in the home cage vs. a clean, novel handling object). We predicted that hut-handled mice would behave similarly to tunnel-handled mice and that mice from both shelter-based handling treatments would display decreased anxiety and increased voluntary interaction with the handler compared to mice in the cupping treatment. These predictions are based on the additional challenges associated with cupping compared to tunnel handling, including its more variable effects on mouse behavior [16] and longer acclimatization times [1,11]. Further, we predicted that mice handled with a familiar shelter would be less anxious and more willing to interact with the handler compared to mice handled with a novel object.

## Methods

All animal procedures were approved by the University of British Columbia’s Animal Care Committee (protocol A23-0228).

### Animals and housing

We obtained 53 experimentally naïve male and female surplus mice slated for euthanasia. These mice were of three different strains: C57BL/6J-*Pbk*^*em1Xxh*^/J (n = 10 males, n = 10 females), C57BL/6J (n = 7 males), and B6N.B6-Tg(Nr4a1-EGFP/cre)820Khog/J (n = 13 males, n = 13 females). All mice were specific-pathogen-free. Daily health assessments were performed by facility animal care technicians, and the experimenters regularly observed for any signs of illness throughout the experimental period. Prior to the experiment, mice had been handled by facility animal care technicians during regular health checks and cage cleanings. While we do not have a precise record of the handling methods for each mouse, mice were most likely tail-handled based on standard handling protocols at the time. At the start of the study, mice were introduced to the experimenters, who consistently used assigned handling methods to ensure a controlled transition into the experimental handling procedures. We were unable to handle one male and one female B6N.B6-Tg(Nr4a1-EGFP/cre)820Khog/J mouse across the first three handling days; these mice were excluded, resulting in a final sample of 51 mice. Our sample was limited by the number of surplus mice available to us, but this sample size is consistent with previous studies assessing handling in mice (n = 10–16 mice per handling treatment) [10,12,21–23].

At the time of testing, mice were 4–18 weeks of age. The mice arrived already marked with ear notches used to identify individuals. Mice were housed in same-sex, same-strain groups of 3–5, consistent with their previous housing, using individually ventilated cages containing aspen chip bedding (Jamieson’s Pet Food Distributors Ltd, Delta, Canada), Enviropak nesting material (Datesand, Stockport, UK) one cotton nestlet (Ancare, Bellmore, USA), and either a clear tunnel (Bio-Serv, Flemington, USA) or a red igloo polycarbonate hut with three entrances (Bio-Serv, Flemington, USA). Food (Lab Diet Rodent Chow 2918) and reverse osmosis tap water were available *ad libitum*. Cages were partially cleaned every two weeks. This process involved transferring mice to clean cages equipped with clean bedding, a new packet of nesting material, a new cotton nestlet, and a new water bottle. The cage lid and food hopper from the previous cage were used, and approximately 2 grams of old nesting material was transferred to the new cage. All data were collected within the two-week interval between routine cage cleanings, ensuring that mice did not undergo any handling outside of the experimental handling sessions. The animal room was kept on a reversed 12 h:12 h light cycle, with lights off from 09:00–21:00. Mice were housed at a mean ± SD room temperature of 21.3 ± 0.1°C and a relative humidity of 47.1 ± 1.1%. At the end of the study, mice were euthanized with isoflurane anesthesia followed by carbon dioxide.

### Experimental design

Pairs of cages (n = 14) were matched by strain, sex, and similarity in age, and home cage shelter (tunnel or hut) was randomly assigned without replacement within each matched pair of cages (n = 7), such that within each pair one cage contained a tunnel and the other a hut. To allow cages of mice to acclimate to their assigned shelter, these were added to existing cages one week prior to the experiment. Within matched-paired cages, mice were randomly assigned without replacement to one of three handling methods: cupping, tunnel, or hut, ensuring that each treatment was tested within each cage.

Mice housed with the shelter type assigned to them were handled using this familiar shelter, and those housed with a different shelter were handled using a clean, novel shelter. For example, if a mouse assigned to hut handling was housed with a hut, then handling would be done with the home cage hut, but if that mouse was housed with a tunnel, then handling would be done with a novel, clean hut. Handling order within cages was assigned randomly in advance and was maintained throughout the experiment.

### Handling sessions

Mice were handled using only their assigned method twice daily for 9 days, for 18 sessions in total. Two individuals conducted the handling sessions, both females with previous mouse handling experience. Handlers alternated daily (i.e., one handler on one day, and the other handler the next day). To facilitate handling, nesting material and home cage shelter (tunnel or hut) were removed from the cage before handling. Using their assigned method, the mouse was transferred to a barren holding cage and was allowed to settle in this cage for 60 s, with the experimenter standing motionless in front of the cage. After 60 s, the mouse was transferred back to their home cage using their assigned method. For cupping, the mouse was gently scooped into the experimenter’s open-cupped hands. To prevent mice from jumping out of the cupped hands, a combination of cupping and tunnel handling was used for the first two handling days [1]: mice were picked up with a novel tunnel and gently tipped backward onto the experimenter’s hand, above the home cage. For tunnel handling, the tunnel was kept stationary at bedding level with one hand, while the other hand gently guided the mouse toward the entrance of the tunnel. Once the mouse was inside the tunnel, the experimenter’s hands were loosely cupped around the openings at either end of the tunnel. For hut handling, the hut was flipped upside down, kept stationary at bedding level and tilted slightly to make it easier for the mouse to walk onto the hut. The mouse was gently guided towards one of the entrances, following the procedure used for tunnel handling. Once the mouse was inside the hut, the experimenter’s hands loosely cupped the entrances of the hut. Each handling session was approximately 3–5 s in length. All sessions were conducted under red lights in the first half of the dark phase of the light cycle, from 10:00–16:00. Between cages, the handler’s gloves and novel handling objects were cleaned with unscented soap, rinsed with water, and dried with a paper towel.

### Voluntary interaction

Willingness to interact with the experimenter was assessed on the first, fifth, and ninth handling days (as described by Hurst & West, 2010 [1]), by which time mice had undergone 1, 9, and 17 handling sessions, respectively. Using their assigned handling method, mice were individually transferred to an empty holding cage to ensure that their responses would not affect cage mates. Mice were allowed 60 s to settle in the holding cage with the experimenter standing motionless in front of the cage. After 60 s, a gloved hand (cupped mice), gloved hand holding a tunnel (tunnel-handled mice), or gloved hand holding an upturned hut (hut-handled mice) was held resting motionless in the front half of the cage floor for 60 s. The durations of the following behaviors were recorded and summed as interaction with the experimenter: sniffing (nose within approximately 0.5 cm of the hand and/or handling object), climbing (all four paws on hand and/or handling object), paw contact (at least one front paw on hand and/or handling object), and inside handling object (all four paws inside tunnel, or hut). After interaction tests, mice were transferred back to their home cage using their assigned handling method.

### Elevated plus maze

Mice were tested in the elevated plus maze (EPM) on the day following the last handling session. The EPM consisted of a white opaque metal maze with two open and two closed arms (all 30.5 x 5 cm with side walls 18.5 cm high on the two closed arms), elevated 25.5 cm above the ground. Using their assigned handling method, mice were placed in the center of the arena, facing an open arm. Mice were allowed to explore the maze for 5 min. After 5 min, mice were returned to their home cage using their assigned handling method, and the maze was cleaned between trials with 70% isopropyl alcohol and a paper towel. All mice were tested under red lights during the dark phase of the light cycle.

### Video analysis

Voluntary interaction and EPM trials were video recorded (Canon VIXIA HF W10, Tokyo, Japan) and scored using Behavioral Observation Research Interactive Software (BORIS, v. 8.27.1) by an observer blind to the familiarity of the handling object, but not to the handling object per se.

All mice (n = 51) were included in the analysis for the voluntary interaction tests. Voluntary interaction tests were scored for time spent sniffing, climbing, paw contact, and being inside of the handling object. For cupped mice, the behaviour ‘inside hand’ was not scored; instead, this was recorded as ‘climbing’. Only one behaviour was scored at a time to avoid overestimating interaction time. These behaviors were summed to calculate the total interaction time with the handler.

Five mice fell off the open arms of the EPM during testing: three from the hut-handled and two from the cupping treatment. These mice were excluded from the analysis, leaving 46 mice in the final analysis for the EPM. EPM tests were scored for time spent in the open arms of the maze.

Twenty-five percent of the voluntary interaction trials and EPM tests were scored by a second blinded observer to calculate interobserver agreement for total interaction time with the handler and the time spent in the open arms of the maze. The Pearson correlation coefficient was calculated as r = 0.98 for total interaction time during the voluntary interaction tests, and r = 0.99 for time spent in the open arms of the EPM.

All trials were scored by MJB, who has several years of experience using BORIS. For interobserver reliability, 25% of videos were scored by an undergraduate student trained by MJB.

### Statistical analysis

Analyses were carried out using SAS OnDemand for Academics (release 3.81, SAS Institute Inc.). A linear mixed-effects model was used to analyze the effects of handling method (i.e., cupping, tunnel, or hut), home cage shelter type (tunnel or hut), and their interaction (i.e., handling object familiarity) on time spent voluntarily interacting with the handler. This interaction term accounted for handling object familiarity, rather than including familiarity as a separate variable. To account for repeated measures, test day was specified as a repeated factor and mouse was specified as subject. Additionally, interactions with voluntary interaction test day were included to account for potential temporal influences on interaction time. Specifically, the model included fixed effects for handling method, home cage shelter type, and voluntary interaction test day, as well as all two- and three-way interactions. A similar linear mixed-effects model was used to analyze these factors and their effects on the time spent in the open arms of the EPM, specifying cage nested within the home cage shelter type as a random factor. Probabilities are presented using Tukey’s adjustment for multiple comparisons. To meet assumptions of normality, total interaction time was winsorized at the 5^th^ and 95^th^ percentiles. Figures were generated in R studio (R version 4.4.2) using the ggplot2 package.

## Results

### Voluntary interaction

Voluntary interaction with the handler varied with handling object (*F*_2_,_123_ = 10.7, *p* < 0.0001): hut-handled mice spent the most time interacting (41.7 ± 1.5 s), followed by tunnel-handled mice (36.1 ± 1.4 s) and then cupped mice (33.0 ± 1.5 s). There was no main effect of home cage shelter type on interaction time (*F*_1,12 _= 0.33, *p* = 0.58), but there was an interaction between home cage shelter type and handling object (*F*_2,123_ = 3.61, *p* = 0.03), indicating that the effect of handling object on voluntary interaction time depended on the familiarity of the object.

Interaction time varied across test days (*F*_2,123_ = 15.3, *p* < 0.0001). There was no interaction between test day and home cage shelter (*F*_2,123 _= 0.22, *p* = 0.80), but there was an interaction between test day and handling object (*F*_4,123_ = 3.00, *p* = 0.02), driven by low interaction times for cupped mice on the first day of testing. Further, there was an interaction between voluntary interaction day and familiarity with the handling object (*F*_4,123_ = 3.04, *p* = 0.02), driven by increased interaction from mice handled with a home cage hut on the first day of testing.

As illustrated in S1 Fig, interaction was generally highest for hut-handled mice, but this effect depended on both familiarity and test day. On day 1, mice handled with a familiar shelter, whether a tunnel or hut, interacted more with the handler. By day 5, this pattern persisted for tunnel-handled mice, whereas hut-handled mice showed the highest interaction when the hut was novel. By day 9, increased interaction was observed among mice handled with both types of novel shelter, suggesting a shift in familiarity effects over time.

**Fig 1:**
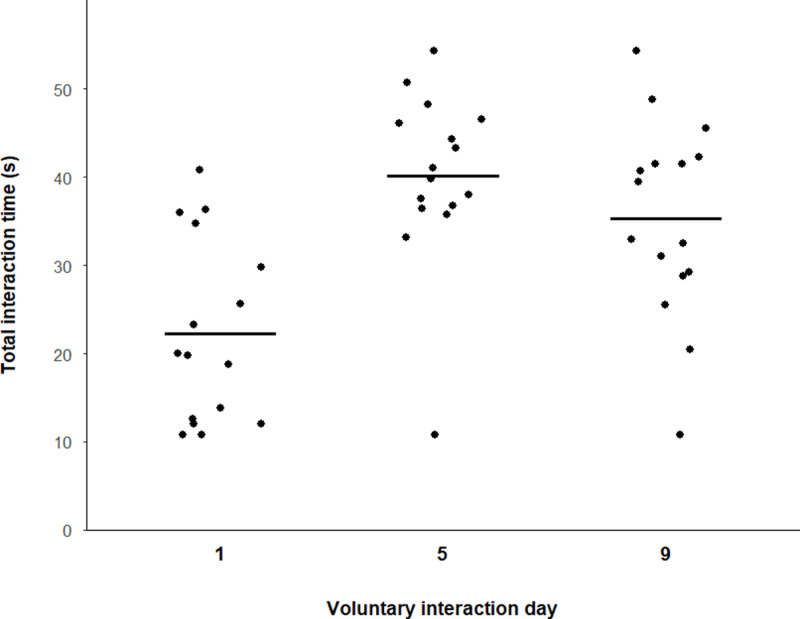
Time spent interacting with the handler (s) per test day for cupped mice. Black bars depict means for each voluntary interaction day. Each point indicates one mouse; points are jittered horizontally to better visualize overlapping points. n = 16 for each voluntary interaction day.

**Fig 2:**
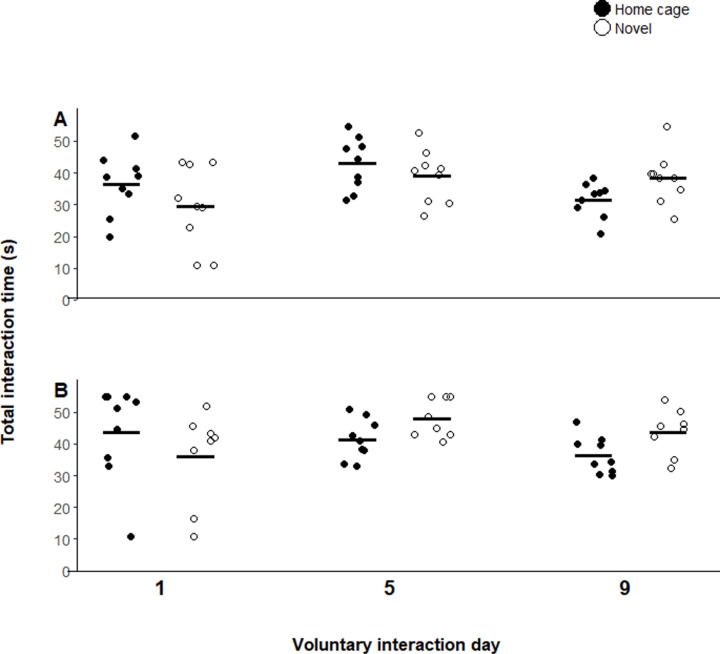
Mean time spent interacting with the handler (s) per test day for tunnel-handled mice **(A)** **and hut-handled mice** **(B)**. Black bars depict means for each voluntary interaction day. Each point indicates one mouse; points are jittered horizontally to better visualize overlapping points. Handling object influenced interaction time (p < 0.0001), with variation across days (p < 0.0001) and an effect of familiarity (p = 0.03). n = 9 for mice handled with a home cage tunnel, novel tunnel, and home cage hut; n = 8 for mice handled with a novel hut.

Across all handling treatments, voluntary interaction was lowest on day 1 and peaked on day 5. Across the three test days, cupped mice interacted with the handler for 22.3 ± 2.6 s, 40.2 ± 2.5 s, and 35.3 ± 2.8 s, respectively (Fig 1). Tunnel-handled mice interacted with the handler and tunnel for 32.9 ± 2.7 s on day 1, 41.0 ± 2.0 s on day 5, and 35.0 ± 1.8 s on day 9, and hut-handled mice interacted with the handler and hut for 40.1 ± 3.6 s on day 1, 44.5 ± 1.7 s on day 5, and 39.8 ± 1.8 s on day 9 (Fig 2).

### Elevated plus maze

**Fig 3:**
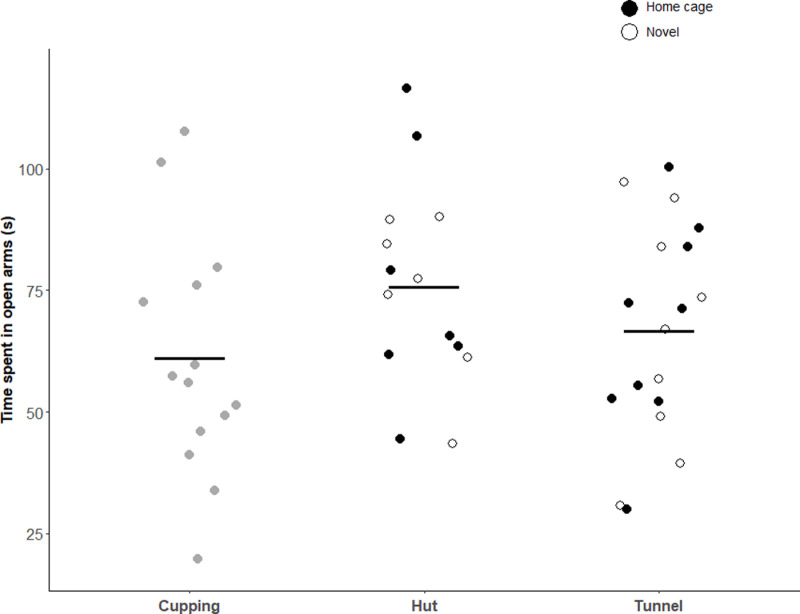
Time spent in the open arms of the EPM (s). Black bars depict means per handling method. Each point indicates one mouse; points are jittered horizontally to better visualize overlapping points. Filled points are for mice handled with their home cage object, while unfilled points are those handled with a novel object. Sample sizes were as follows: cupping (n = 14), handled with novel hut (n = 7), handled with home cage hut (n = 7), handled with a novel tunnel (n = 9), handled with home cage tunnel (n = 9).

Time spent in the open arms of the EPM varied between individuals, but there was no effect of handling object (*F*_2,28_ = 1.84, *p* = 0.18) or handling object familiarity (*F*_2,28_ = 0.03, *p* = 0.97) on this measure (Fig 3). Mice in the cupping treatment spent (mean ± SE) 64.6 ± 6.3 s in the open arms. Mice handled with a novel or familiar hut spent 76.5 ± 8.8 s and 79.2 ± 8.8 s in the open arms, respectively. Those handled with a novel or familiar tunnel spent 68.2 ± 8.1 s and 68.1 ± 8.1 s in the open arms, respectively.

## Discussion

Handling mice with cupped hands or with a tunnel is known to result in less anxiety and improved interaction with the handler compared to tail handling [1–5,8,11,12,16,24]. Other objects commonly found in mouse cages may be used for handling, such as huts. Wallin (2023) [20] found that, compared to tail-handled mice, mice handled with their home cage hut displayed reduced anxiety in the open field test and improved voluntary interaction with the handler. The current study is the first to assess huts for mouse handling in comparison with the established refined alternatives of cupping and tunnel handling. Further, we investigated whether the familiarity of the handling object (home cage or novel) influenced voluntary interaction with the handler and anxiety, assessed via time spent in the open arms of the EPM.

We found considerable variation in responses to handling, with some evidence that mice prefer interacting with huts over cupped hands and tunnels. Across all voluntary interaction days, hut- and tunnel-handled mice interacted with the handler more than did cupped mice. Both objects offer additional surface area to explore and provide a physical barrier between the mouse and handler. Compared to tunnels, upturned huts offer extra surface area that allows for exploration, such as climbing and rearing within the hut, which are not possible inside a tunnel.

A potential explanation for the increased interaction observed in tunnel-handled versus cupped mice is that mice have a natural tendency to seek narrow spaces for hiding [1]. However, this preference for an enclosed space cannot explain that interaction was highest for hut-handled mice, as huts were upturned during handling. The natural preference of mice for enclosed spaces should be considered when selecting handling objects. For example, future studies should consider using huts with solid floors, or other more enclosed designs that allow mice to hide.

We suggest that the decreased interaction with mice in the cupping treatment was because this handling method involves direct contact with the experimenter’s gloved hands. This interaction may present a less predictable and less rigid surface, also potentially carrying novel or aversive scents [25].

Consistent with Gouveia and Hurst (2019) [11], we found no effect of handling object on time spent in the open arms of the EPM. By the time mice were tested in the EPM, they had experienced handling for nine days. This period was likely sufficient to familiarize mice with their assigned handling method, as evidenced by comparable interaction times observed on the final test day.

Cupped mice showed the highest variability in voluntary interaction across days. This result is in line with prior studies showing that cupping was associated with variable responses in behavioral testing [16]. Previous work found that mice took longer to habituate to cupping than tunnel handling and that for both treatments, willingness to interact with the handler increased across multiple days [1,11,16]. We found that by day 5 of handling, the interaction time of cupped mice had nearly doubled, reaching levels similar to those of tunnel- and hut-handled mice. Habituation reduces rodent aversion to human contact [1,26,27]. Similarly, Hurst and West (2010) [1] noticed improved interaction with the handler among cupped mice after 5 days of handling. These results add to existing evidence that, while mice can be habituated to cupping through additional handling sessions, this method can result in less consistent responses [11,16].

We found that handling mice with a familiar object improved voluntary interaction during early handling sessions. Over multiple handling days, this difference was less apparent, suggesting that mice learned to recognize the ‘novel’ handling objects. Both tunnel- and hut-handled mice initially spent less time interacting with a novel versus familiar object, likely due to the novel scent, as suggested by Gouveia & Hurst (2013) [2]. However, interaction patterns differed between handling objects over time. Hut-handled mice showed a faster shift toward increased interaction with the novel hut, while tunnel-handled mice maintained a preference for the familiar tunnel for longer. This may indicate that hut-handled mice adapted to novelty more quickly than tunnel-handled mice. Gouveia & Hurst (2013) [2] investigated the effect of using tunnels that were shared between cages on mouse anxiety and willingness to interact with the handler. Unlike the present study, which involved cleaning handling objects between each cage, their protocol used a shared tunnel for mice of the same sex and strain. They found that interaction with a shared tunnel increased across handling days, and mice handled by a familiar versus shared tunnel showed similar levels of anxiety in the EPM [2]. Given the conflicting evidence regarding the effectiveness of cleaning agents in removing pheromones [28,29], the potential impact of residual scents from the cleaning process should be considered when interpreting voluntary interaction results. Consistent with their findings, our results indicate that object familiarity influenced interaction time most strongly in early sessions but became less relevant as handling experience increased. By the fifth handling day, differences in interaction times between mice handled with a novel versus familiar object had diminished, and both groups showed similar levels of anxiety in the EPM.

Our findings indicate that using an upturned hut to handle mice is a viable alternative to the established refined handling methods of cupping and tunnel handling. In laboratories, the choice of method will depend on practical constraints like accessibility and ease of use. Huts may be a practical alternative to tunnels especially in facilities that already use huts in mouse cages.

Further work is required to determine how handling objects affect the time taken to handle mice, especially during routine procedures like cage cleanings. Previous research found that some mice will voluntarily enter the tunnel [1,30], and in the current study, we also noted that some mice voluntarily entered the hut, facilitating handling. An added advantage of upturned huts is that they allow for simultaneous transfer of multiple mice, potentially enhancing the efficiency of handling.

Using an upturned hut as a handling object may increase the risk that animals can escape during handling events. One approach to avoid escape is to gently restrain animals by the base of their tail while they are supported in the hut. Hurst & West (2010) [1] reported that mice initially handled using cupped hands or a tunnel did not find brief restraint and lifting of their hind legs by the tail for abdominal inspection aversive. Future research should document the risk of escape, if this can be reduced by modifying existing handling practices, and how these modifications affect mouse welfare.

A limitation of the present study is that we used just one type of hut and one type of tunnel. Huts can vary and the design may affect how mice respond to these shelters as handling objects. We used a red, polycarbonate, dome-shaped hut with three entrances. In contrast, Wallin (2023) [20] used a cardboard hut that differed from ours in size, shape, and number of entrances. Plastic may facilitate handling by allowing mice to be easily tipped out, but mice may prefer the textured surface of cardboard that may offer better grip, as suggested by Wallin (2023) [20]. Huts were likely designed with a focus on how mice would use these as shelter; if huts (or other cage furnishings) are to be used for handling, then research is also required to ensure that both needs are well served. Selecting a hut design that is optimal for handling but poor as a shelter would represent an unfortunate compromise for mouse welfare.

Similar to previous studies [11,16,20], mice underwent brief handling sessions for 9 days. However, other studies have shown that mice can be habituated to handling objects even when handled very briefly and infrequently (e.g., 2-s handling sessions during fortnightly cage cleaning [11,16]). Future studies should assess whether mice can be habituated to hut handling following a similar schedule.

Several factors may affect mouse responses to handling, such as strain and past interactions with humans. Previous work has shown that anxiety varies by strain [31–33] and sex [34]. Tunnel handling can reduce anxiety across sexes, regardless of the light or dark phase of the diurnal cycle and the previous experience of handlers [1,2]. However, some studies have found strain-specific behavioral responses to tunnel handling [2,30,35]. A strength of the present study is that we considered a diverse group of mice, but we were unable to control factors such as previous experiences with handling. Mice with a history of negative interactions with humans may be more fearful and require more time to acclimate to handling. In the present study, mice were surplus and likely tail-handled prior to the experiment, which could have influenced initial interaction, particularly for cupped mice where direct contact with the handler was required. However, interaction increased across handling days, suggesting that mice adapted to their assigned handling methods over time. Future studies should consider tracking prior handling experiences to better understand their effects on responses to different handling techniques.

## Conclusions

Hut-handled mice spent more time interacting with the handler compared to those who were tunnel-handled and even more than those who were cupped. Our findings show that handling mice with a familiar home cage shelter is preferable to an unfamiliar one, but habituation to handling with a novel shelter is achievable by the fifth day of handling. We recommend hut handling as an effective and practical alternative to cupping and tunnel handling, particularly in facilities that already have access to huts.

## Supporting information

S1 Fig Interaction effects between handling object, familiarity, and voluntary interaction test day. Mean voluntary interaction time across test days for tunnel-handled and hut-handled mice, shown by object familiarity (home cage (HC) vs. novel). Error bars represent SEM.
(TIF)

S1 Video Cupping.
(MP4)

S2 Video Tunnel handling.
(MP4)

S3 Video Hut handling.
(MP4)
