# Supplementary figures and images for: The effects of handling on mouse behavior: cupped hands versus familiar or novel huts or tunnels
Source: PLoS One. 2025 May 19;20(5):e0323785. doi: 10.1371/journal.pone.0323785 (PMC12087983; doi:10.1371/journal.pone.0323785)

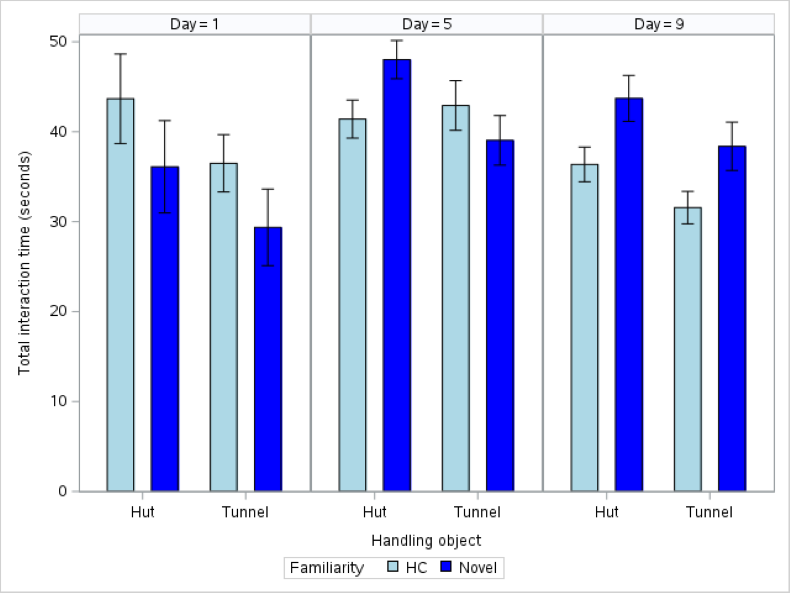

Supplement: S1 Fig — Mean voluntary interaction time across test days for tunnel-handled and hut-handled mice, shown by object familiarity (home cage (HC) vs. novel). Error bars represent SEM. (TIF) [file pone.0323785.s001.tif]
